# Coinfection by *Aspergillus* and Zygomycetes Species in a Case of Acute Rhinosinusitis

**DOI:** 10.1155/2011/382473

**Published:** 2011-09-12

**Authors:** Dhara Vaidya, Parul Shah

**Affiliations:** Department of Microbiology, Smt N. H. L. Municipal Medical College, Ellisbridge, Ahmedabad 380006, India

## Abstract

Invasive mycotic infections can be effectively treated if rapid identification of fungus is obtained. We reported a case of coinfection by *Aspergillus* and *Rhizopus* sp. involving nose, paranasal sinuses, orbit, and brain in a 68-year-old known hypertensive male. He was presented to ENT OPD with history of fever and intermittent headache since fifteen days along with history of right-sided nasal obstruction and proptosis since seven days. CT scan of brain and paranasal sinuses showed findings of pansinusitis with cellulitic changes in right orbit. MRI confirmed the same along with features of intracranial extension with focal meningitis in right frontotemporal region. Laboratory parameters did not conclude much except for leucocytosis and hyponatremia. Patient was taken for endoscopic debridement from nose and paranasal sinuses, and tissue was sent for microbiological and histopathological examination. Minced tissue was processed, and after 48 hrs of incubation two types of growth were identified, one was yellowish, granular, and powdery consistent with *Aspergillus* sp., and another was cottony and woolly consistent with *Rhizopus* sp. LCB mount confirmed presence of *Aspergillus flavus* and *Rhizopus arrhizus*. Patient responded to therapy with IV amphotericin B and surgical debridement. On discharge patient's condition was good.

## 1. Introduction

 Fungal infection of paranasal sinuses is an increasingly recognized entity both in normal and immunocompromised individuals. A variety of different causative organisms are responsible for paranasal mycosis, *Aspergillus* and Zygomycetes being the commonest [1]. 

 Paltauf [2] first identified rhinocerebral mycosis in 1885. He described a case of rhinocerebral mucormycosis. Aspergillosis and zygomycosis are the commonest causes of central nervous system mycosis [3]. Paranasal mycosis manifests as two distinct entities, a benign or noninvasive infection and more serious invasive infection which occurs in immunocompromised individuals and characterized by its rapid onset, ability to invade tissues, and cause of destruction. Early diagnosis is vital in these infections because delay in initiation of treatment can be life threatening due to propensity of fungi to invade adjacent blood vessels and to embolize to distant organs including brain. Invasive form of fungal sinusitis is usually associated with poorly controlled diabetes but any immunocompromised patient is at risk from this opportunistic infections [1]. In rhinocerebral mycosis the disease originates in the nasal/sinus mucosa after inhalation of fungal spores and takes a rapidly progressive course by extending to neighboring tissues including orbit and central nervous system [3]. 

 Rhinocerebral mycosis carries high residual morbidity and mortality due to angioinvasive property of fungi causing vascular occlusion and extensive tissue necrosis. Impaired delivery of antifungal drugs to the site of infection because of vascular thrombosis and limited aggressive surgery due to complex anatomy of the rhinoorbitocerebral regions cautions for early diagnosis and aggressive management in these patients [4].

## 2. Case Report

 A 68-year-old Hindu male, who was a known case of hypertension since 10 years, reported to us with history of low-grade fever and intermittent headache since 10 days. Headache was generalized and relieved with medication. Meanwhile patient developed c/o of right-sided nasal obstruction and proptosis since seven days. There was no history of nasal discharge/bleeding or any ear/throat pain or any discharge.

 He was admitted in local hospital for the same for 3 days; here he was diagnosed as having pneumonia with right lower zone consolidation. Antibiotics and symptomatic treatment were given. There was no improvement in his condition so he was referred to our institution for confirmed diagnosis and management. 

 Patient had a past history of CV stroke followed by facial palsy 5 years back. Patient was nondiabetic but was on regular antihypertensive drugs. He did not have any history of chronic illnesses or surgery in the past. Patient had an agricultural background.

 On examination patient was afebrile with normal pulse and heart rate. All other systems were normal. On ENT examination right maxillary sinus tenderness was present. Local examination of nose showed no local deformity, and vestibules were normal. Ophthalmological examination revealed right-sided proptosis with restricted ocular movements in all directions. Conjunctival congestion with pseudophakia was present. Pupil on right side was nonreactive to light. All findings suggested 3rd and 6th nerve palsy.

 Laboratory investigations showed leucocytosis with total count of 15,800/cumm. Peripheral smear did not reveal any abnormality. Patient was hyponatremic on admission. On routine macroscopic and microscopic examination, CSF was turbid with high protein count of 75 mg/dL, and total cells were 4 cells/uL, all being lymphocytes. Patient was negative for HIV. CT scan of brain and paranasal sinuses showed mucosal thickening of all sinuses with cellulitic changes in right orbit involving extraconal, intraconal, and preseptal compartment of orbit. MRI scan suggested changes of pansinusitis with possible fungal infection in right posterior ethmoid, sphenoid, and maxillary sinuses. There was inflammatory phlegmon with early developing abscess in right medial orbit with erosion of lamina papyracea and involvement of medial orbital content with extension of infection into infratemporal fossa and intracranial extension with focal meningitis in right frontotemporal region. Provisional diagnosis based on clinical and radiological findings was acute invasive rhinosinusitis with right eye proptosis and involvement of central nervous system.

 The patient underwent endoscopic surgery under general anaesthesia. Fungal debris was removed from both nasal cavity and paranasal sinuses. Intraoperatively blackish mass along with necrotic tissues was removed.

**Figure 1:**
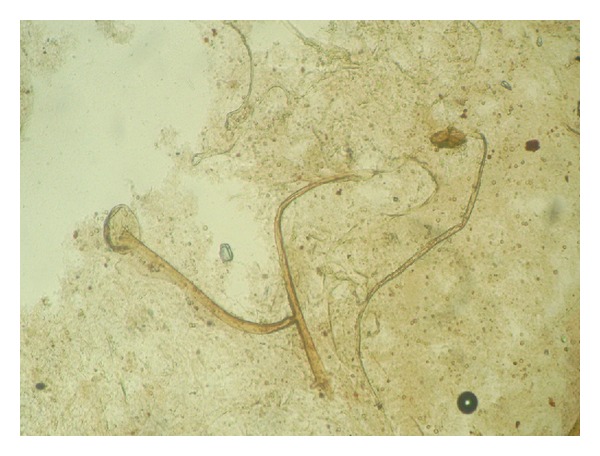
Direct wet mount preparation of tissue showing nodal sporangiophore with umbrella-shaped appearance of sporangium (40x).

**Figure 2:**
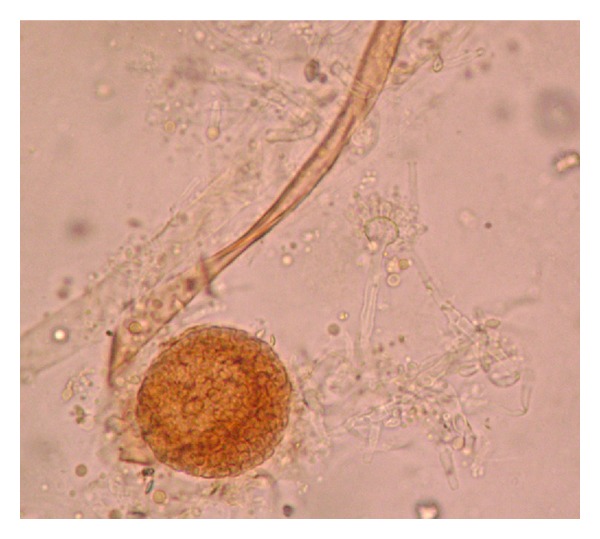
Thin, septate hyphae with vesicle and conidia along with aseptate hyphae and sporangium in KOH preparation from tissue (40x).

**Figure 3:**
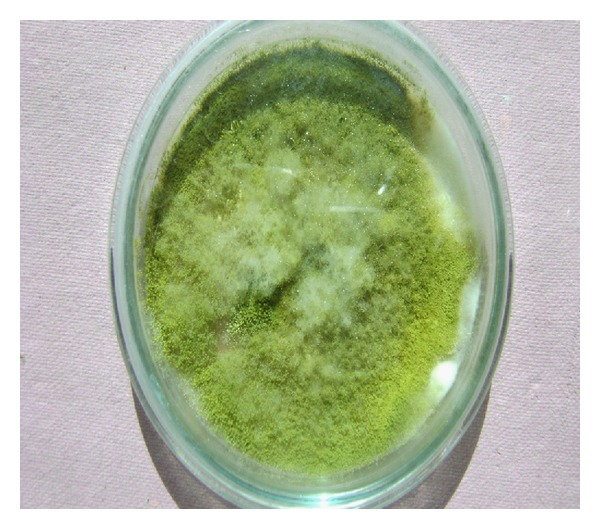
SDA plate on the 5th day showing white, cottony, fluffy growth of Zygomycetes species superimposed on powdery, granular growth of *Aspergillus* species.

**Figure 4:**
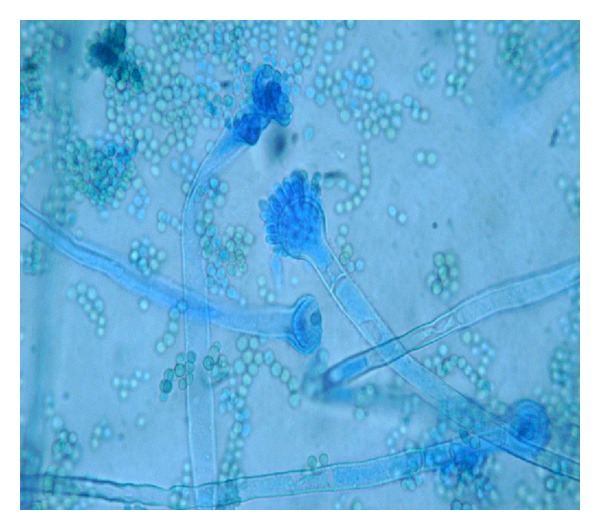
LCB preparation from mixed culture showing ruptured sporangium with developing vesicle in the same field (40x).

 Clinical specimens were collected and sent to the laboratory for microbiological and histological examination. On gross examination, tissue was brown to black in colour, necrotic, and hemorrhagic. The tissue, after mincing into small pieces, was subjected to 10% potassium hydroxide (KOH) mount which showed two different types of fungal elements. There were narrow, branched, septate hyphae along with globose vesicle containing phialides and conidia. Another type of hyphae was wide, aseptate, and ribbonlike with sporangium containing round sporangiospores. To our surprise, umbrella-shaped empty sporangia along with underdeveloped rhizoids and nodal sporangiophores were also visible in wet mount preparations (Figure 1). Thin, septate hyphae with vesicle and conidia along with aseptate hyphae and sporangium in KOH preparation were seen in the same field (Figure 2). The minced tissue specimen was inoculated on SDA plate with antibiotics for fungal culture in duplicate; one set was incubated at 25°C and another at 37°C. On SDA plate, and after 48 hrs 2 types of growth were observed. One was mat-like initially having rugose texture which changed to granular and powdery with yellowish-green surface pigment; another was cottony, woolly, and fluffy (Figure 3). Subculture from both types of growth was performed for isolation of both fungi. Microscopic examination with lactophenol cotton blue staining also confirmed 2 different types of fungus. There were hyaline, branched, septate hyphae with large globose vesicle containing uniseriate phialides fully covering the vesicle with chains of yellowish-green round conidia with foot cells attached at the conidiophores. LCB preparation from mixed culture showing ruptured sporangium with developing vesicle in the same field in Figure 4. All these findings favor diagnosis of *Aspergillus flavus. *Another finding was broad, aseptate, irregularly branched ribbonlike hyphae with sporangiophores arising from the hyphae and enlarging distally into hemispherical columellae with flattened base containing round sporangia. Sporangiospores were ovoid to elliptical and light brown in colour. At some places umbrella-shaped ruptured sporangia along with underdeveloped internodal rhizoids were seen confirming the diagnosis of *Rhizopus arrhizus.*

**Figure 5:**
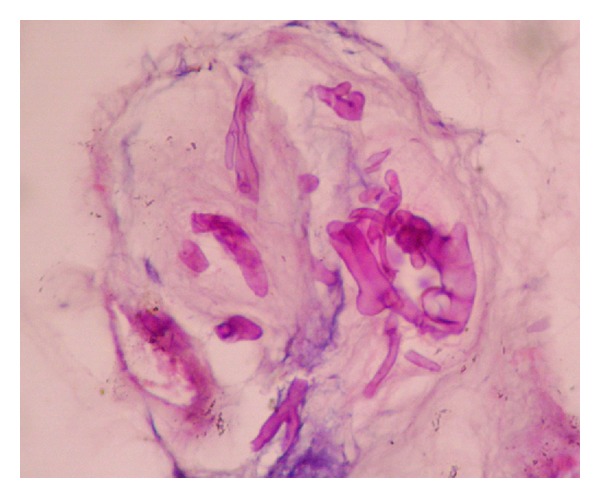
PAS staining of tissue section showing thin, dichotomous hyphae intermingled with wide, irregular aseptate hyphae (100x).

 The fungus was identified as coinfection of *Aspergillus flavus* and* Rhizopus arrhizus*. The histopathological report endorsed our findings showing broad and aseptate hyphae suggestive of Zygomycetes sp. and thin, septate hyphae with dichotomous branching suggestive of *Aspergillus* sp. (Figure 5).

## 3. Discussion

 Zygomycosis and aspergillosis are two serious opportunistic infections that are commonly seen in immunocompromised patients. Since both these fungi invade the vessels of the arterial system, an early and rapid diagnosis by direct examination of KOH mounts of the relevant clinical sample can confirm the diagnosis [5]. 

*Aspergillus* is the most common fungus in histologically verified CNS mycosis from India, and presents with focal neurological signs and symptoms. Zygomycosis is infrequently reported from India and the rhinocerebral form is the commonest form of zygomycosis [6]. *Aspergillus* spreads to the CNS by direct inoculation by trauma or surgery or direct extension from paranasal sinuses or eye or invasion of arteries and veins and fungemia. Zygomycosis is caused by direct extension from paranasal sinuses. Infection begins in superior turbinates and spreads to paranasal sinuses, orbit, and brain after inhalation of sporangiospores [7].

*Aspergillus* is a saprophytic fungus, that is, ubiquitous throughout the world; it causes infection following inhalation of *Aspergillus* conidia or mycelial fragments on vegetation, decaying matter, and soil. *Aspergillus* causes allergic bronchopulmonary aspergillosis, fungus ball, invasive aspergillosis, paranasal granuloma, and endocarditis [8]. Zygomycosis is a progressive infection caused by one of the phycomycetes. There are large, thin-walled, and nonseptate fungi. Zygomycetes consist of two orders—Mucorales and Entomophthorales, which contain genera and species of medical importance. Fungi of order mucorales are distributed into six families (Mucoraceae, Cunninghamellaeceae, Saksenaeaceae, Thamnidiaceae, Syncephalastraceae, and Marsileaceae) and cause mucormycosis. Species belonging to the family Mucoraceae are more commonly isolated from patients with mucormycosis than of any other family. Among the family Mucoraceae,* Rhizopus arrhizus* is by far the most common cause of infection [9, 10].

 The classification scheme proposed by Meltzer et al. [11] is primarily intended to guide clinical research and divides rhinosinusitis into four categories: acute presumed bacterial rhinosinusitis, CRS without polyps, CRS with polyps, and classic allergic fungal rhinosinusitis. This classification system includes information about the type of infection (viral, bacterial, and fungal), complications, inflammatory markers, and radiologic findings to categorize patients. The more complex system allows the subdivision of patients into more detailed subgroups to determine the precise target of the new intervention or medication being studied.

 In our case, patient is a 68-year-old hypertensive male with no other significant history pointing towards immunocompromised state. Patient was admitted with history of fever and intermittent headache along with nasal obstruction and proptosis developed in course of time. This type of presentation typically correlates with features of rhinocerebral mycosis in which the disease originates in the nasal/sinus mucosa after inhalation of fungal spores and takes a rapidly progressive course by extending to neighboring tissues including orbit and CNS. The initial clinical findings in orbital involvement are most commonly headache, lethargy, and facial pain. These may be rapidly followed by restriction of ocular movements, proptosis, ptosis, and periorbital cellulitis. Paralysis of the 3rd, 4th, and 6th cranial nerves and visual loss due to central retinal artery occlusion is not uncommon [4]. 

 In a review of 929 cases of zygomycosis by Roden et al., sinus (39%) is the most common form of zygomycosis [12]. *Rhizopus* is the most often suspected etiological agent. Overall mortality is 55%, and coinfection with *Aspergillus* sp., proven or probable, was noted in 44% cases. Highest survival rate is with surgery combined with antifungal agents [13].

 Only one case of combined zygomycosis and aspergillosis at a single site, that is, the oropharyngeal region, has been reported from a patient with Castleman's disease [14]. Binder and Ruchel described a case of combined infection with *Aspergillus* and Zygomycetes sp. involving the lungs, spleen, and the brain in a patient of acute myeloid leukemia and leading to fatal outcome inspite of early antimycotic treatment [15].

 While CT of the sinuses is more sensitive for bony changes, MRI provides superior evaluation of intracranial and intraorbital extension of disease and should be included as part of the initial evaluation. We could not provide the picture of CT image. Aggressive surgical debridement and empiric systemic antifungal therapy, followed by serial endoscopic evaluations, are the mainstays of treatment [16]. Contrast MRI of the brain and sinuses should be done in all patients with suspected rhinocerebral mycosis. Contrast enhancement, extension of the disease into the orbit and cranial cavity can be well delineated on MRI. Same wise in our patient CT and MRI proved a major role in diagnosis.

 With prior availability of amphotericin B, mucormycosis was almost universally fatal. With early diagnosis and a combination of amphotericin and radical debridement of infected tissues, fatality rate has dropped. Debridement should be started as early as possible when the gangrenous form of infection is detected. Mortality is high in patients with rhinocerebral mycosis if the treatment is delayed. However survival depends significantly on recovery of immune function [17]. Our patient responded well to amphotericin B, which is fungistatic for the agents of zygomycosis and is the only US Food and Drug Administration-approved drug for the initial therapy of invasive zygomycosis [18]. But the gold standard therapy for our case is prompt surgical debridement with removal of all fungal debris followed by antifungals.

 In conclusion, as no effective chemoprophylactic regimen is available for prevention of mucormycosis and invasive aspergillosis, preventive strategies include limiting the sources of contamination in the environment of patients at risk and careful monitoring. Finally, prompt diagnosis and aggressive treatment of a potentially fatal condition can be achieved with heightened awareness and better cooperation between clinicians, microbiologists, and pathologists.
